# Epidemiology of Brucellosis and Q Fever in Linked Human and Animal Populations in Northern Togo

**DOI:** 10.1371/journal.pone.0071501

**Published:** 2013-08-12

**Authors:** Anna S. Dean, Bassirou Bonfoh, Abalo E. Kulo, G. Aboudou Boukaya, Moussa Amidou, Jan Hattendorf, Paola Pilo, Esther Schelling

**Affiliations:** 1 Department of Epidemiology and Public Health, Swiss Tropical and Public Health Institute, Basel, Switzerland; 2 University of Basel, Basel, Switzerland; 3 Centre Suisse de Recherches Scientifiques en Côte d’Ivoire, Abidjan, Côte d’Ivoire; 4 Ecole Supérieur d’Agronomie, Université de Lomé, Lomé, Togo; 5 Direction de l’Elevage, Ministère de l’Agriculture, de l’Elevage et de la Pêche, Lomé, Togo; 6 Directional Régional de la Santé - Savanes, Dapaong, Lomé, Togo; 7 Institute for Veterinary Bacteriology, University of Bern, Bern, Switzerland; Texas A&M Health Science Center, United States of America

## Abstract

**Background:**

Although brucellosis (*Brucella* spp.) and Q Fever (*Coxiella burnetii*) are zoonoses of global importance, very little high quality data are available from West Africa.

**Methods/Principal Findings:**

A serosurvey was conducted in Togo’s main livestock-raising zone in 2011 in 25 randomly selected villages, including 683 people, 596 cattle, 465 sheep and 221 goats. Additionally, 464 transhumant cattle from Burkina Faso were sampled in 2012. The serological analyses performed were the Rose Bengal Test and ELISA for brucellosis and ELISA and the immunofluorescence assay (IFA) for Q Fever Brucellosis did not appear to pose a major human health problem in the study zone, with only 7 seropositive participants. *B. abortus* was isolated from 3 bovine hygroma samples, and is likely to be the predominant circulating strain. This may explain the observed seropositivity amongst village cattle (9.2%, 95%CI:4.3–18.6%) and transhumant cattle (7.3%, 95%CI:3.5–14.7%), with an absence of seropositive small ruminants. Exposure of livestock and people to *C. burnetii* was common, potentially influenced by cultural factors. People of Fulani ethnicity had greater livestock contact and a significantly higher seroprevalence than other ethnic groups (Fulani: 45.5%, 95%CI:37.7–53.6%; non-Fulani: 27.1%, 95%CI:20.6–34.7%). Appropriate diagnostic test cut-off values in endemic settings requires further investigation. Both brucellosis and Q Fever appeared to impact on livestock production. Seropositive cows were more likely to have aborted a foetus during the previous year than seronegative cows, when adjusted for age. This odds was 3.8 times higher (95%CI: 1.2–12.1) for brucellosis and 6.7 times higher (95%CI: 1.3–34.8) for Q Fever.

**Conclusions:**

This is the first epidemiological study of zoonoses in Togo in linked human and animal populations, providing much needed data for West Africa. Exposure to *Brucella* and *C. burnetii* is common but further research is needed into the clinical and economic impact.

## Introduction

Brucellosis is one of the most common zoonotic diseases globally [1]. *Brucella* spp are transmitted to people through the consumption of unpasteurised dairy products or direct contact with infected animals, particularly abortion materials [2], [3]. This bacterial disease causes not only a severely debilitating and disabling illness [4], but also has major economic ramifications due to time lost by patients from normal daily activities [2] and losses in animal production [5]. High quality incidence data for human brucellosis are predominantly available from the Middle East and North Africa region [6] with no high quality data published from West Africa. Exposure to *Brucella* has been demonstrated in cattle populations in West Africa [7]–[10], with *B. abortus* isolated from bovine hygromas in Mali [10] and the Gambia [7].

In recent years, *Coxiella burnetii* has become a bacterial zoonosis of increasing interest, due to an unprecedented, large-scale outbreak of Q Fever in the Netherlands from 2007–2010 with approximately 4,000 notified human cases [11]. The most common route of infection for people is the inhalation of dust contaminated by infected animal fluids, followed by ingestion, particularly of unpasteurised dairy products [12], [13]. The global importance of Q Fever is unknown. In West Africa, there is evidence of exposure to the organism amongst fever patients in Mali [14], but more representative and comprehensive seroprevalence and clinical data are needed.

A One Health approach to disease prevention and control not only fosters collaboration between human and animal health sectors, but also brings additional benefits to each [15]. The economic benefits include those generated through the sharing of resources such as vehicles or field equipment [16], [17], or through a reduced burden on the health care system by controlling diseases in the animal reservoir [5], [18]. The simultaneous assessment of exposure in people and animals provides a more complete picture of the disease epidemiology, deepening our understanding of disease risk and providing an important knowledge base for policy development. In resource-poor settings, an integrated human-animal health system is particularly needed in order to increase the cost effectiveness of disease prevention and control interventions, improve the efficiency of disease surveillance and response, and strengthen the health system as a whole [19].

In Togo, animal brucellosis was first documented in the 1960s [20]. Although bovine brucellosis seroprevalence studies [21] and *Brucella* sp. identification [22] were conducted in the 1980s and early 1990s, simultaneous assessment of exposure patterns in people and livestock was lacking. The Savannah Region is Togo’s principal livestock-raising zone. Being the northernmost of five administrative regions, it is bordered by Ghana to the west, Burkina Faso to the north, and Benin to the east. Cross-border livestock movements are an inherent component of livestock management practices, via largely unregulated trade routes or semi-nomadic movements known as transhumance [23]. Official policy for transhumance in West Africa was agreed upon in 1998 by member countries of the Economic Community of West African States (ECOWAS). Togo permits the entry of foreign herds in search of pasture and watering points from January to May of each year. The Savannah Region of Togo is, therefore, a diverse epidemiological setting, with its livestock disease status likely to be linked to that of neighbouring countries.

## Methods

### Ethics Statement

This study was approved by the Ethics Committee for Health Research (Comité de Bioéthique pour la Recherche en Santé) of the Ministry of Health of Togo (ref. 0131/2011/MS/CAB/DGS/DPLET/CBRS) and the Livestock Division (Direction de l’Elevage) of the Ministry of Agriculture, Livestock Production and Fisheries of Togo. In Switzerland, approval was given by the Ethics Commission of the Cantons of Basel-Stadt and Basel-Land (Ethikkommission beider Basel) (ref. 146/10) and the Research Commission of the Swiss Tropical and Public Health Institute of Basel, Switzerland. Informed written consent was obtained from all literate participants or, in the case of minors, from their parents or guardian. For illiterate participants, informed consent was recorded with a fingerprint, which was witnessed and documented in writing by a literate acquaintance of the participant. Obtaining consent in this way was approved by the aforementioned ethics commissions. The collection of blood samples from animals was always supervised by a veterinarian. Animals were handled in manner aiming to minimise stress and suffering, and were only restrained for the shortest period of time necessary.

### Village Serosurvey

**Figure 1:**
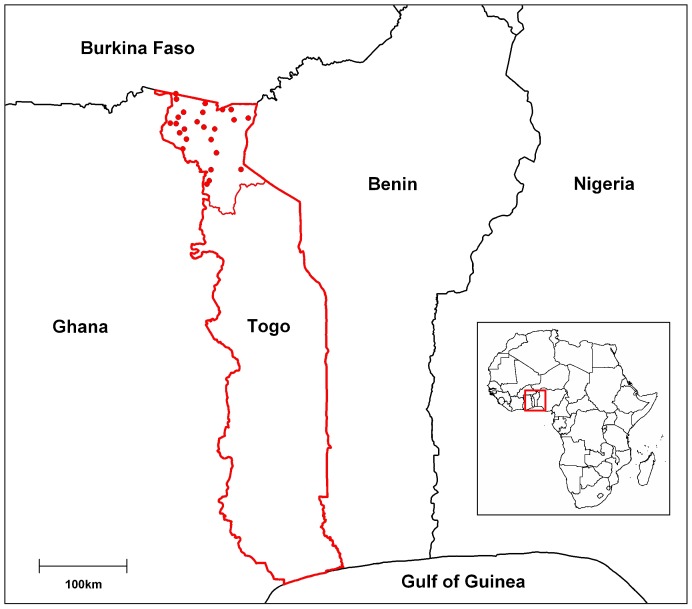
Map of study site. The national borders of Togo are shown in red and the boundary of the Savannah Region is indicated by the dashed red line. The red points correspond to the 25 randomly selected villages included in the survey.

Using a detailed regional map and MapInfo Professional® 7.0 SCP, 25 geographical coordinates were randomly selected from the Savannah Region, excluding national parks where settlements were prohibited. The 25 villages closest to each coordinate were visited in May – June 2011 and enrolled in the study after approval from the village chief (see Figure 1). A day was allocated for the study team to return to conduct the survey in each village. The study team included nurses from local health centres, as well as veterinary technicians and veterinarians. Cluster sample sizes were calculated using the formula of Bennett [24] with 95% confidence intervals according to Newcome [25].

#### Household and participant selection

Due to the absence of a household list, a central point in the village was identified and a direction selected by spinning a pen. The number of houses from the centre to the edge of the village was counted in this direction, and a number randomly selected between 1 and the total number of houses. The household corresponding to this number was identified by counting the doors in this direction, moving away from the central point. The study was explained in the local language and one adult and one child aged between 5–14 years of age were enrolled by randomly selecting from those household members willing to participate. The household with its door closest to the first selected household was also visited and a further two participants enrolled. The team then returned to the centre of the village to choose a second direction. An intraclass correlation coefficient of 0.1 and an expected brucellosis seroprevalence of 3% [26] were assumed. A sample size of 15 people from 25 villages would achieve a precision corresponding to a 95% confidence interval not wider than 6 percentage points. Consequently, the sampling procedure above was repeated until a total of 7–8 households were enrolled in each village, i.e. 14–16 people.

The majority of cattle herds in northern Togo are managed by people of Fulani ethnicity, pastoralist communities who represent the most dispersed ethnic group in West Africa [27], [28]. Fulani communities represent only a very small proportion of the overall population of each village, and their households are often clustered at the edge of village. As the household selection procedure described above for the general population would not have provided an adequate sample size from the Fulani communities of each village, targeted sampling was conducted. Approval was first sought from the Fulani chief and the community members were then gathered together to explain the aims and methods of the study. Participants were selected from those willing to participant by random number allocation. The planned sample size for the Fulani was 14–16 people, thus totalling 28–30 people per village (half of which were Fulani).

Blood was collected from people by a trained nurse, in parallel with data relating to the presence of specific symptoms during the previous month (fever, headache, night-time sweats, sleeping difficulties, or muscle, joint or nerve pain). Basic data relating to demography (age, sex, ethnicity) and risk factor exposure were collected through structured interviews with participants or their parent/guardian. The risk factors assessed were the consumption of dairy products (from any species) that had not been subjected to heat treatment (boiling), and contact with either cattle or small ruminants, where contact was defined as living in a household that owned or looked after cattle or small ruminants.

#### Animal sampling

In each village, households owning cattle and/or small ruminants (sheep, goats) were identified through discussion with the village chief, the Fulani chief, and the local veterinary technician. In each village, these households were visited by the study team and invited to participate. Owners were requested in advance to keep their animals nearby their homes on the pre-selected sampling day. As a full census of the village’s livestock population was not available, sampling was distributed across all identified herds by dividing the total desired number per village by the number of herds/flocks to obtain the number of animals to be randomly sampled per herd/flock. Animals under 3 months of age were excluded. An intraclass correlation coefficient of 0.1 [29] and an expected brucellosis seroprevalence of 5% in cattle and 1% in small ruminants [26] were assumed. A sample size of 20 cattle and 25 small ruminants from 25 villages would achieve a precision corresponding to a 95% confidence interval not wider than 7 percentage points for cattle and 4 percentage points for small ruminants.

Blood collection was performed by a veterinarian and veterinary technician. Individual and herd data (age, sex, herd size, abortions during previous year, and abortions during reproductive life for transhumant cattle only) were obtained through structured interviews with herd owners or herdsmen.

### Transhumant Serosurvey

In February – March 2012, information about the location of transhumant cattle herds was obtained from key informants, including local livestock owners and herders, Fulani communities, and animal health personnel. Given the available resources and time period, a sample size of 15 herds was deemed to be realistic, with 25 animals per herd based on an expected brucellosis seroprevalence of 5% [24], [25]. Blood collection from cattle as well as structured interviews with herdsmen were conducted as per the village survey. Blood samples were not collected from the herdsmen.

### Serological Analyses

After collection, all blood samples were immediately chilled on ice and centrifuged the same day. Serum was then collected and frozen at −20°C until analysis. Sera from the village survey were transported frozen according to international biosafety standards to the Swiss Tropical and Public Health Institute in Basel, Switzerland for serological analysis. Sera from the transhumant survey were analysed at the Central Veterinary Laboratory of the Ministry of Agriculture, Livestock and Fisheries in Lomé, Togo, and training was provided to the laboratory technicians. A sub-sample of the sera tested in Switzerland was re-tested in Togo for comparison, to ensure an agreement of results by the two laboratories.

#### Brucellosis

All human samples were tested by the Rose Bengal Test (RBT) (Brucella Rose Bengale, Bio-Rad Laboratories, CA, USA), using a serum:reagent ratio of 1∶1 for 8 minutes. Positive sera were sequentially diluted with saline to obtain dilutions from 1/2 to 1/16, then retested as per the protocol of Diaz et al [30]. Additionally, all sera were tested by indirect IgG ELISA. Samples positive by IgG ELISA or RBT were also tested by IgM ELISA (Serion ELISA classic Brucella IgM/IgG, Institut Virion\Serion GmbH, Würzburg, Germany), in order to identify recent infections. Samples were classified as positive or negative according to the manufacturer’s recommended cut-off ranges and doubtful samples were re-tested.

All animal samples were tested by the RBT, using a serum:reagent ratio of 1∶1 for cattle and 3∶1 for small ruminants for 4 minutes, as per recommendations of the World Organisation for Animal Health (OIE) [31] . Additionally, all samples were tested by indirect ELISA (CHEKIT Brucellose Serum ELISA Test Kit, IDEXX Laboratories, ME, USA) and classified as positive or negative according to the manufacturer’s recommended cut-off ranges. Samples positive by RBT but negative by ELISA were tested by the Complement Fixation Test according to OIE standards at the Institute for Veterinary Bacteriology, University of Bern.

#### Q Fever

A random sub-sample of human sera was tested using an indirect IgG Phase II ELISA (*Coxiella burnetii* ELISA IgG, Vircell SL, Granada, Spain). Samples were classified as positive or negative according to the manufacturer’s recommended cut-off ranges and doubtful samples were re-tested. In order to assess the suitability of the ELISA cut-off value for the Togo samples, the immunofluorescent antibody test (IFA) for Phase II IgG was performed by Vircell S.L. in Spain (*Coxiella burnetii* IFA IgG, Vircell SL, Granada, Spain) [32].

A random sub-sample of sera from village livestock was tested using an indirect IgG ELISA (CHEKIT Q-Fever Antibody Test Kit, IDEXX Laboratories, ME, USA). Samples were classified as positive or negative according to the manufacturer’s recommended cut-off ranges and doubtful samples were re-tested.

### Hygroma Fluid Sampling and Culture

In March 2012, cattle with joint hygromas from study villages and neighbouring villages were identified through discussion with cattle owners or herders and local veterinary technicians. Fluid samples from joint hygromas were collected from nine cattle, six of which were from herds sampled within the village serosurvey. All hygromas were involving the carpus, except for one over the hock. After aseptic collection of joint fluid, a sterile swab tip was soaked in the fluid and sealed in liquid Amies transport medium. Two swabs were harvested per animal. The swabs were stored at 4°C before transportation to Switzerland, according to international biosafety standards, for microbiological culture at the Institute for Veterinary Bacteriology, University of Bern.

The swabs were cultivated on tryptic soy agar plates supplemented with 5% sheep blood (BD, NJ, USA) and on Brucella-selective agar (BSA). The BSA contained Brucella-medium base (Oxoid, Basingstoke, UK), 5% inactivated horse serum and 7.5 mL modified Brucella Selective Supplement (Oxoid, Basingstoke, UK). The plates were incubated for 10 days at 37°C in 5% CO_2_. Colonies from pure cultures were suspended in lysis buffer (0.1 M Tris-HCl pH 8.5, 0.005% Tween 20, 0.24 mg/mL Proteinase K) and incubated for 60 minutes at 60°C and for 15 minutes at 95°C. The lysates were then filter sterilised.

Identification of the genus *Brucella* was performed by realtime PCR according to the protocol of Hinic et al [33]. Multiple Loci Variable Number of Tandem Repeats Analysis (MLVA) was carried out over 16 loci (panel 1, panel 2A and panel 2B markers) for the isolated strains and for *B. melitensis* strain 16M^T^, as previously described [34], [35]. The PCR products were analysed with the Agilent 2100 Bioanalyzer using a DNA 1000 LabChip kit (Agilent Technologies, Waldbronn, Germany) according to the manufacturer's protocols, and compared with the results of De Santis et al [10]. Some of the PCR products were sequenced in order to confirm the exact size of the amplicons. The MLVA data were analysed using the *Brucella* aggregated database hosted on MLVAnet (http://mlva.u-psud.fr/) developed by University Paris Sud in Orsay, France [36].

### Data Analysis

Data were entered in duplicate into an Access 2003 database (Microsoft, USA), in order to detect data entry errors, and compared using the Data Compare function of Epi Info 3.5.3 (Centers for Disease Control and Prevention, USA). All statistical analyses were performed in Stata 10.1 (StataCorp LP, USA).

As animals from the same village were observed to regularly mix at common grazing and watering points, the clustering level for livestock was considered to be the village rather than the household. The apparent seroprevalence was calculated using Generalised Estimating Equations (GEE) with a binomial distribution and an independent correlation structure. To adjust for test performance, a range of plausible values for sensitivity and specificity were specified (presented in the Results section) from which a value was randomly sampled for the calculation of an adjusted seroprevalence. The model was run for 1,000 simulations, with the random selection of sensitivity and specificity for each simulation. The mean adjusted seroprevalence was then determined.

Mixed effects logistic regression was used to calculate odds ratios to assess covariates such as demographic characteristics and potential risk factors for exposure, as well as the relationship between human and livestock seroprevalence at the village level. To assess serological test performance for brucellosis, the level of agreement between the ELISA and RBT was determined by calculating the inter-rater agreement between two unique raters. For human Q Fever, ELISA sensitivity and specificity were calculated using IFA as the gold standard reference test [12].

## Results

### Human Serosurvey

From the 25 villages, 683 people participated in the survey, of which 255 (37.3%) were of Fulani ethnicity. The age and sex distribution are shown in Table 1. Consumption of non-boiled dairy products was common, particularly amongst Fulani with almost all adults (95.6%) reporting consumption compared to less than two thirds (60.7%) of the rest of the population, hereafter referred to as non-Fulani. Most households reported owning or looking after cattle, sheep, or goats, with 96.9% Fulani and 83.4% non-Fulani households reporting this livestock contact. Nearly three quarters (73.9%) of Fulani men listed livestock as a principal source of income, as opposed to 13.8% non-Fulani.

**Table 1:** Characteristics of study participants from 25 villages in northern Togo.

People				
Ethnicity	n	Male	Age in years	
			Median (range)	≤15
Fulani	255	118 (46.3%)	25.5 (5–85)	95 (37.3%)
Non-Fulani	428	242 (56.7%)	26 (5–90)	195 (45.6%)
*Total*	683	360 (52.7%)	26 (5–90)	290 (42.5%)
**Animals**				
**Species**	**n**	**Male**	**Median age in years (range)**	**–**
Cattle: from villages	596	201 (33.7%)	6 (0.3–16)	–
Cattle: transhumant*	464	93 (20.0%)	2 (0.5–15)	–
Sheep	465	52 (11.2%)	3 (0.3–12)	–
Goats	221	24 (10.9%)	2 (0.3–12)	–

* Transhumant cattle were sampled from 18 herds across 11 sites.

#### Brucella seroprevalence

Seroprevalence results for *Brucella* spp. are presented in Table 2. Only five people were positive by IgG ELISA and three people by RBT. One person was positive to both tests. Of the three people positive by RBT, only one remained positive after sequential serum dilution. This person was also IgM ELISA positive. Overall, six of these 7 seropositive people were of Fulani ethnicity. The apparent seroprevalence for Fulani was 2.4% (95%CI: 1.0–5.6), compared to 0.2% (95%CI: 0.0–1.6) for the rest of the population, as shown in Table 2. Seropositive participants were re-assessed by the study team for symptoms compatible with active brucellosis. These participants were not clinically unwell at the time of examination and none had a clinical history supporting active disease, including the IgM seropositive participant. They were provided with information about the disease and were monitored by their local nurse.

**Table 2:** Seroprevalence of brucellosis (*Brucella* spp) and Q Fever (*Coxiella burnetii)* in people and livestock.

Brucellosis			
Population	n	Seroprevalence (%) (95%CI)	
		Unadjusted	Adjusted for ELISA performance
Humans: Fulani	255	2.4 (1.0–5.6)	**–**
Humans: non-Fulani	428	0.2 (0.0–1.6)	**–**
Cattle: from villages	596	9.2 (4.3–18.6)	8.9 (7.0–10.7)
Cattle: transhumant	464	7.3 (3.5–14.7)	7.1 (5.0–9.5)
Sheep	465	0	–
Goats	221	0	–
**Q Fever**			
Humans: Fulani	134	45.5 (37.7–53.6)*	**–**
Humans: non-Fulani	144	27.1 (20.6–34.7)*	**–**
Cattle: from villages	242	15.7 (11.6–20.9)	14.5 (10.3–19.0)
Sheep	207	16.4 (11.4–23.1)	15.0 (11.1–20.3)
Goats	198	7.1 (3.3–14.6)	6.6 (3.5–9.6)

* Using IFA cut-off of 1∶32 for serological evidence of exposure.

#### *C. burnetii* seroprevalence

Two hundred and seventy-eight human sera (134 Fulani and 144 non-Fulani), representing 40.7% of the total sample, were randomly selected for IFA and ELISA. Serological evidence of exposure to *Coxiella burnetii* was common, with 100 people having Phase II IgG titres by IFA ranging from 1∶32 to 1∶256, in 23 of 25 villages. Assuming IFA to be the gold standard for Q Fever diagnosis (i.e. 100% sensitivity and specificity), the seroprevalence in non-Fulani was 26.9% (95%CI: 20.4–30.6%) and 45.3% (37.6–53.3%) in Fulani using a cut-off value of 1∶32 as evidence of serological exposure [37]. The odds of being seropositive was 2.3 times higher (95%CI: 1.4–3.8) for Fulani compared to non-Fulani when adjusted for age and sex. Only 7 people had titres of 1∶256 which, as a single Phase II IgG titre, could suggest recent or active infection [38]. However, none of these cases reported symptoms during the previous month that clinically supported such a diagnosis. Owning or looking after livestock was associated with a greater odds of being seropositive using an IFA cut-off value of 1∶32, although the 95% confidence intervals included 1. The odds ratios for seropositivity and contact with cattle or small ruminants were 1.6 (95%CI: 0.9–2.9) and 1.3 (95%CI: 0.7–2.3), respectively. Consumption of non-boiled milk did not increase the odds of seropositivity, with an odds ratio of 1.1 (95%CI: 0.5–2.3).

Using the manufacturer’s recommended ELISA cut-off value, the sensitivity and specificity of the Phase II IgG ELISA were 84.8% and 63.0%, respectively, for evidence of serological exposure (IFA titre of 1∶32). However, for IFA titres of 1∶256, which can be considered the threshold for recent or active infection, sensitivity increased to 100% and specificity to 69.4%.

### Animal Serosurvey

From the 25 villages, 596 cattle, 465 sheep, and 221 goats were randomly sampled. Additionally, serum was collected at 11 different sites from 464 transhumant cattle of 18 herds originating from Burkina Faso. Owners of transhumant cattle at two sites refused to participate due to time constraints. The age and sex distribution of animal subjects are shown in Table 1.

#### Brucella serology

Seroprevalence results for livestock are presented in Table 2. Cattle seropositive to *Brucella* spp by indirect IgG ELISA or RBT were only detected in half of the villages (13 of 25). The apparent seroprevalence by ELISA using the manufacturer’s recommended cut-off value was 9.4% (95%CI: 4.6–18.4%), whereas the seroprevalence by RBT was lower at 5.7% (95%CI: 2.4–12.8%). Based on conservative estimates of indirect ELISA sensitivity and specificity of 94–98% and 97–99% respectively [26], [39], the seroprevalence adjusted for ELISA performance was 8.9% (95%CI: 7.0–10.7%).

In transhumant cattle, the apparent seroprevalence was slightly lower. The seroprevalence was 7.3% (95%CI: 3.5–14.7%) by ELISA and 4.5% (95%CI: 2.4–8.5%) by RBT. The seroprevalence adjusted for ELISA performance was 6.2% (95%CI: 4.4–8.2%). Overall, the two tests had an agreement of 95.9%.

The odds of being seropositive by ELISA increased with age. When adjusted for age, cows had an odds of *Brucella* seropositivity that was 5.3 times higher (95%CI: 1.5–18.7) than bulls. Results of the univariate and age-adjusted analysis of predictors of brucellosis seropositivity are shown in see Table 3. Seropositive cows had a 3.8 times greater odds (95%CI: 1.2–12.1) of having aborted a foetus during the previous year than seronegative cows, when adjusted for age.

**Table 3:** Predictors of seropositivity to *Brucella* spp and *Coxiella burnetii* by ELISA in cattle.

Bovine Brucellosis (*Brucella* spp*)*					
Predictor	Number of subjects(proportion seropositive)		Odds ratio (95%CI)		
			Univariate Analysis	Adjusted for sex	Adjusted for age
11 yrs–16 yrs*°	48 (18.0%)		22.9 (7.2–73.3)	16.2 (4.9–53.4)	–
6 yrs–10 yrs*°	378 (12.2%)		5.7 (2.9–11.3)	4.4 (2.2–8.8)	–
3 mths–5 yrs*	613 (20.8%)		–	–	–
Cows	766 (11.2%)		9.8 (2.9–32.9)	–	5.3 (1.5–18.7)
Bulls	294 (1.0%)		–	–	–
Abortion during previous year+	32 (31.3%)		4.1 (1.4–12.3)	–	3.8 (1.2–12.1)
**Bovine Q Fever (** ***Coxiella burnetii)***					
Abortion during previous year+		8 (16.0%)	6.3 (1.2–33.2)	–	6.7 (1.3–34.8)

* Excluding animals for whom age was not known (n = 21).

°The reference category for the odds ratios is 3 mths –5 yrs.

+ Excluding cows under 3 years of age.

All small ruminants were negative by indirect IgG ELISA. Eleven were positive by RBT but were negative by CFT. Thus, the apparent seroprevalence in sheep and goats was 0%.

#### *C. burnetii* serology

For testing by indirect IgG ELISA, 242 cattle, 207 sheep, and 198 goat sera were randomly selected. In contrast to *Brucella* spp, *C. burnetii* seropositivity (meaning at least one seropositive animal) was detected in almost all villages (24 of 25). In these 24 seropositive villages, the mean village-level seroprevalence was 14.9% (range: 3.6–37.5%).

The apparent seroprevalence by ELISA using the manufacturer’s recommended cut-off value was 16.1% (95%CI: 8.8–21.5%) in cattle, 16.2% (95%CI: 11.1–23.0%) in sheep, and 8.8% (95%CI: 4.4–16.9%) in goats, using a GEE model with clustering at the village level. Based on conservative estimates of ELISA sensitivity and specificity of 90–94% and 97–99% respectively [26], [39], adjustment of seroprevalence for test performance estimated seroprevalences to be 14.8% (95%CI: 10.8–19.2%) in cattle, 14.4% (95%CI: 10.6–19.1%) in sheep, and 8.3% (95%CI: 4.2–12.4%) in goats.

Cows that had aborted a foetus during the previous year had an odds of being *C. burnetii* seropositive that was 6.7 times higher (95%CI: 1.3–34.8) than those that had not aborted (Table 3), when adjusted for age. This relationship was not evident for small ruminants. Sex and age were not predictors of seropositivity. There was only a very weak linear correlation between the village-level seroprevalence of people and livestock, with an adjusted R-squared value of 0.05.

### Hygroma Fluid Culture

Although there was no growth on the BSA plates, mixed bacterial cultures grew on blood agar. *Brucella*-like colonies were then purified onto blood agar plates and isolates were recovered from hygroma samples from three cows, all of which were seropositive by RBT and ELISA. The genus *Brucella* was confirmed by realtime PCR. Assessment by MLVA-8 (panel 1) identified the isolates as *B. abortus.* Each isolate had a distinct Variable Number of Tandem Repeats (VNTR) profile by MLVA-16 (panels 1, 2A and 2B). The full biotyping results will be presented in detail in a separate publication.

## Discussion

This is the first study of brucellosis and Q Fever in linked animal and human populations in Togo. Simultaneous assessment of the exposure of people and animals provides a more complete epidemiological picture and deepens our understanding of infection patterns at the animal-human interface. The findings of this study are relevant not only for Togo, but also for neighbouring countries in West Africa, given the frequent cross-border movement of livestock often occurring outside official channels.

Most published high quality data on the incidence of human brucellosis in Africa comes from northern Africa [6], where *B. melitensis* has an important impact on human and animal health [40]. The findings of this study suggest a different disease epidemiology in northern Togo, with no evidence of circulation of *B. melitensis*. Despite the ownership/management of livestock and consumption of non-boiled dairy products being common, brucellosis does not appear to pose a major human health problem in the study zone. The disease may, however, still have a significant economic impact through a reduction in cattle productivity. The distinct VNTR profiles of the three *B. abortus* isolates obtained from the culture of bovine hygroma fluid suggests that the organism has been circulating in the study zone for some time. Cows that were seropositive for *Brucella* spp. or *C. burnetii* were more likely to have aborted a foetus during the previous year. Indeed, the Food and Agricultural Organization of the United Nations (FAO) estimated in 2002 that milk and meat production in West Africa could increase by US$56–168 million/year if brucellosis were eliminated [41].

Due to the absence of a perfect test, *Brucella* serology is notoriously difficult to interpret. All serological tests have limitations when used as screening tools [42]. In this study, the apparent seroprevalence has been adjusted for the performance of the serological tests and the influence of age and sex. However, it remains only an approximation of the true disease seroprevalence in the population. Exposed but seronegative animals, for example, are not captured in the adjusted seroprevalence estimates. The OIE guidelines for brucellosis state that both the RBT and ELISA are suitable screening tests for the control of brucellosis at the local or national level. Positive reactions should be tested by a confirmatory strategy, such as the CFT [31], [42]. Given that the 11 small ruminants in this study that were seropositive by RBT and seronegative by ELISA remained negative when tested by CFT, the ELISA may be a more specific test than the RBT for brucellosis. It may also have a higher sensitivity, with more cattle diagnosed seropositive by ELISA than by RBT. However, an additional confirmatory test would be required in order to investigate this further. The levels of seropositivity of village and transhumant cattle were similar. Given the frequent cross-border movements and mixing of livestock in West Africa, these findings suggest that the livestock disease status in one country may be potentially linked to that of its neighbour.

Exposure of animals and people to *C. burnetii* was common, although the proportion of seroconversions representing clinical disease is not known. More research is needed into identifying appropriate cut-off values for diagnostic tests in endemic settings. However, given that 5.0% of 870 acute febrile patients presenting to health centres in Tanzania were diagnosed with acute Q Fever [43], it is indeed likely that the pathogen causes substantial morbidity in Togo.

Cultural practices may influence zoonotic disease risk. The seroprevalence of both diseases were higher amongst the Fulani compared to the rest of the population, possibly reflecting their greater ownership or management of livestock and their increased consumption of non-boiled dairy products. However, a positive association between the consumption of raw milk and seropositivity to *C. burnetii* could not be demonstrated in this study. The role of milk consumption in *C. burnetii* transmission is not fully understood, with asymptomatic seroconversion without clinical illness reported [44], [45].

### General Recommendations

This is the first joint human and animal zoonotic disease study in Togo, which is an important component of the broader One Health concept. A One Health approach to zoonotic diseases research should be encouraged in Togo, as it may guide disease prevention and control strategies, bring economic benefits to both sectors and strengthen the health system as a whole [15].

Community awareness of practices risking exposure to zoonotic diseases is needed. Given their higher levels of exposure, this is particularly important for Fulani communities. Boiling milk before consumption would reduce the risk of exposure to foodborne pathogens in general. The importance of avoiding contact with aborted materials, as well as basic hygiene practices including handwashing and environmental cleanliness [46], should also be communicated. Given the low levels of literacy amongst study participants, appropriate methods include verbal communication of health messages by the local nurse or the village and Fulani chiefs, or the use of visual tools. In order to successfully engage communities, the support of both the village chief and the Fulani chief is essential. However, information, education and communication campaigns are costly to implement. These costs would need to be assessed in relation to the expected combined benefits for human health and livestock productivity [18].

Diagnosing and treating zoonotic diseases is particularly challenging in malaria-endemic countries [47], [48]. The screening of fever cases for differential diagnoses, including Q Fever, should be carried out in order to determine their role in febrile illness [14], [43]. This would first require training of health workers and laboratory staff, and strengthening of laboratory capacity. In terms of animal health, the higher odds of abortion by seropositive cows suggests that livestock productivity is affected by both brucellosis and Q Fever. Further investigation into the economic impact of these diseases would allow the identification and tailoring of cost-effective control strategies, such as vaccination. These strategies must be assessed in the context of overall benefits to public health, the livestock production sector, and household economies [5]. There may be opportunities to combine the selected interventions with other livestock health programs, such as the anthrax vaccination campaigns which take place annually in the study zone.

### Study Limitations

There are several limitations to this study. Firstly, the proportion of the total population sampled in each location is unknown, due to the unavailability of recent, comprehensive census data. The age distributions of the human and animal study populations are unlikely to reflect the true age distribution of the general population. Secondly, the village survey was conducted during the transition period between the dry and wet seasons (May-June 2011), before road access became too difficult due to flooding. In some particularly dry areas, livestock left the village early in the morning in search of watering points and grazing pasture. These animals were not captured by the sampling strategy of the field team. Given the practicalities of conducting a field survey in remote, rural communities, this potential source of selection bias was unfortunately unavoidable. There may have also been temporal variations in pathogen exposure and clinical symptoms that were not captured by the cross-sectional study design. Finally, more detailed information about risk factors, including the dairy products consumed and the level of contact with livestock and abortion materials, would have allowed a more thorough assessment of exposure risk.

### Conclusions

This is the first epidemiological study of brucellosis and Q Fever in linked human and animal populations in Togo, providing much needed data for the West African region. *Brucella* spp and *C. burnetii* are shown to be circulating in the study zone, but further research is needed into their clinical and economic impact. Education of the community about practices risking exposure to zoonotic diseases is needed.
